# Accelerating cancer without mutations

**DOI:** 10.7554/eLife.45809

**Published:** 2019-03-21

**Authors:** Douglas E Brash

**Affiliations:** 1 Department of Therapeutic RadiologyYale School of MedicineNew HavenUnited States; 2 Department of DermatologyYale School of MedicineNew HavenUnited States; 3 Yale Cancer CenterYale School of MedicineNew HavenUnited States

**Keywords:** ultraviolet radiation, melanoma, modifier genes, cancer, ribosomes, RNA-binding protein, Mouse

## Abstract

Mice get melanoma faster when they have common, inherited variants in a few genes that control cell-wide changes but also respond to the environment.

**Related research article** Ferguson B, Handoko HY, Mukhopadhyay P, Chitsazan A, Balmer L, Morahan G, Walker GJ. 2019. Different genetic mechanisms mediate spontaneous versus UVR-induced malignant melanoma. *eLife* **8**:e42424. doi: 10.7554/eLife.42424

For researchers, cutaneous melanoma is a conundrum. This deadly skin cancer arises from melanocytes, the cells that give our skin its color. It is tightly linked to sun exposure, yet the cancerous lesions often appear in places usually covered by clothes, and in people who are only intermittently in the sun. However, individuals who have experienced repeated sunburn, especially as a child, are more at risk. Overall, it is still unclear how these cancers emerge. Inherited genes – including those that guide skin pigmentation and DNA repair – explain a mere 18% of melanoma risk (Hulur et al., 2017).

It is difficult to study melanomas in humans in a controlled manner, so researchers have genetically engineered several strains of mice that can develop the disease (Day et al., 2017). Some of these strains get melanomas as adults after being exposed to a single dose of ultraviolet (UV) light early in life, resembling the way that adult humans are more likely to get skin cancers after multiple sunburn events as a child. Since UV light damages the DNA, it could potentially cause the genetic mutations that drive healthy cells to become cancerous, but studies have shown that this is rarely the case when mice receive a single early UV exposure (Mukhopadhyay et al., 2016). The UV dose could also create epigenetic changes – that is, modifications in how genes are expressed – but these do not force future mutations. So what is this single UV exposure doing?

Now, in eLife, Grant Morahan, Graeme Walker and colleagues – including Blake Ferguson as first author – report having pinpointed a small number of unexpected, normal genes that fast track the onset of either spontaneous or UV-induced melanomas (Figure 1; Ferguson et al., 2019). First, the researchers, who are based at the QIMR Berghofer Medical Research Institute, the Harry Perkins Institute of Medical Research and Edith Cowan University, selected a strain of mice genetically modified with two mutations found in human patients so the rodents can develop melanomas (including in response to an early UV dose). These animals were then crossed with 70 strains from a community resource, the Collaborative Cross. Mice from this resource have genetic backgrounds that contain a diverse collection of apparently normal variants of murine genes, often termed 'modifier genes' (Morahan et al., 2008). The offspring therefore all carried the two mutations that made them prone to melanomas, but each mouse also had different modifier genes inherited from its Collaborative Cross parent.

**Figure 1.**
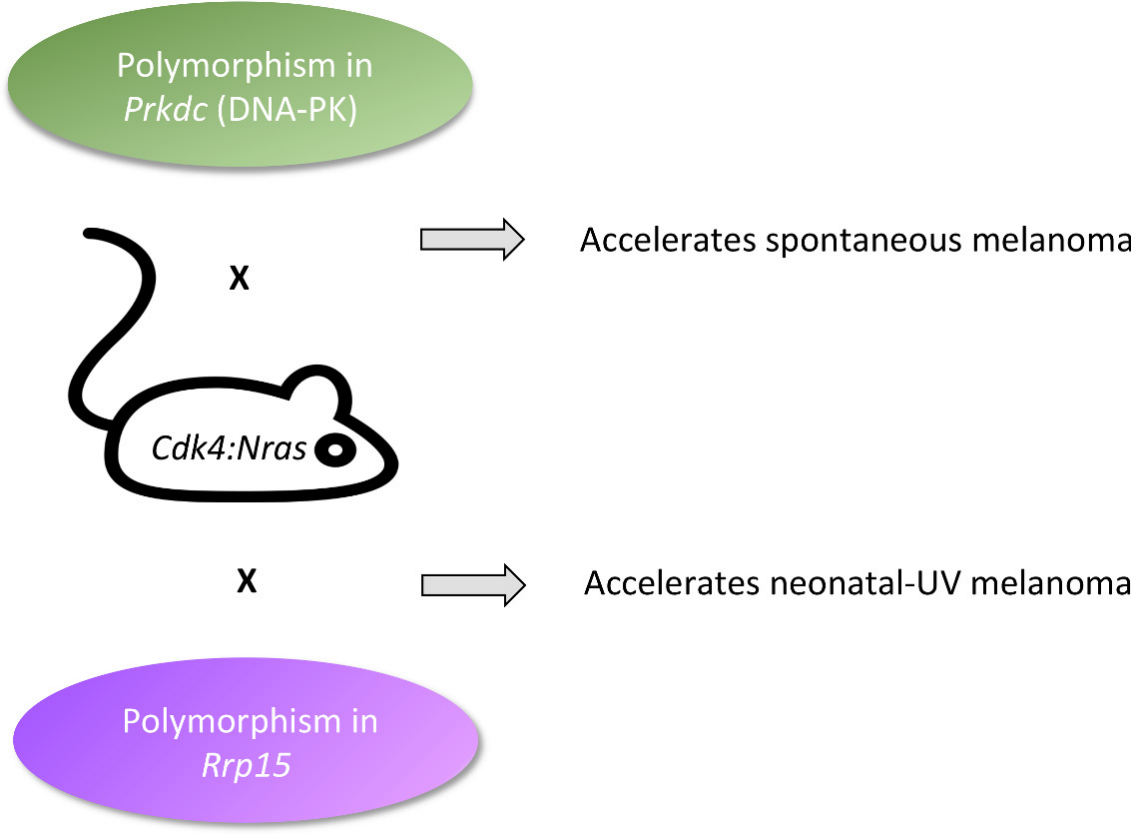
Fast-tracking melanomas. The onset of melanoma in *Cdk4:Nras* mice can be accelerated by modifier genes normally present in the genetic background of other mouse strains. Normal variants in *Prkdc* (green, top) made spontaneous melanomas appear faster. This gene codes for a protein that is well-known for repairing DNA strand breaks, and less well-known as an RNA binding protein that chemically modifies proteins involved in transcription, receptor signaling, and cytokine synthesis. Normal variants in *Rrp15* (purple, bottom) made neonatal UV melanomas appear more quickly. This gene codes for an RNA-binding protein that is involved in cell cycle arrest, apoptosis, and cell migration and invasion. Because UV turns on the *Rrp15* gene, this result highlights how the environment can lead to cancer by triggering large-scale physiological changes that precede, rather than follow, ‘driver’ mutations.

Spontaneous melanomas appeared more quickly in mice with variations in a DNA region that contained eight genes with mutations in their regulatory regions. One of these genes, *Prkdc*, emerged as a strong candidate because it carried two deleterious mutations. It codes for DNA-PK, an enzyme that repairs DNA double-strand breaks. Such breaks can arise if cells have difficulty replicating their genetic information before dividing. Failing to repair them leads to changes in the number of gene copies (which is often seen in cancer). Moreover, several genes with a similar DNA repair role are known tumor suppressors. In a human skin tissue bank, the researchers found that the expression of *PRKDC* was correlated with that of genes contributing to this repair mechanism. Curiously, its expression correlated even better with genes that participate in RNA-binding.

Melanomas that appeared after a single neonatal UV exposure were accelerated by changes in a specific DNA region on a different chromosome. These included missense mutations in *Rrp15*, the only gene in that region whose inheritance tracked the accelerated melanomas, and whose expression changed in the skin after UV exposure. The variants in *Rrp15* did not impair DNA repair or enhance the migration of melanocytes or inflammatory cells, showing that they do not alter normal cellular responses in a way that could obviously lead to cancer. Unexpectedly, this gene codes for a protein present in ribosomes, which belongs to a family of RNA-binding proteins intimately involved in building these structures.

In fact, ribosomes have recently come under the spotlight as being more than workbenches for translating mRNA into protein. First, the specific RNA-binding proteins in a ribosome choose which mRNAs get translated into protein (Shi et al., 2017). In addition, RNA-binding proteins have signaling roles in the cell: deleting *Rrp15* changes how the cell divides, kills itself or migrates into neighboring tissue (Xu et al., 2016; Dong et al., 2017). More broadly, disturbing the regulation of ribosomal proteins at the gene or protein level is associated with cells becoming cancerous, including in human melanomas (Xu et al., 2016; Hayward et al., 2017). Indeed, many proteins and pathways that are linked with cancer control the synthesis of ribosomal RNA, and are in turn regulated by ribosomal proteins (Quin et al., 2014; Xu et al., 2016).

These new roles add meaning to the observation that, in the context of spontaneous melanoma, the expression of *PRKDC* correlates with genes that code for RNA-binding proteins. Indeed, DNA-PK, the protein that *PRKDC* codes for, can also bind to RNA and has many other roles in the cell besides DNA repair. For instance, it intervenes on pathways linked to inflammation, skin differentiation, hormone responses, the control of the cell cycle, and the building of ribosomes. The prominent role of RNA-binding proteins in accelerating melanoma suggests we should reassess how they participate in early onset of cancer.

Based on this, Ferguson et al. draw two conclusions. Firstly, that somatic mutations – like the two present in the genetically engineered mice – were necessary but not sufficient for rapid cancers; the missing impetus came from normal germline variants in RNA-binding proteins. The number and diversity of such modifier genes means that any particular one may be found in only a few tumors, but when it comes to gene variants, frequency should not be confused with impact (Cannataro et al., 2018). Secondly, the team discovered that the formation of ribosomes was a major gene network upregulated by neonatal UV exposure. Therefore, they propose that global physiological events occurring hours after the single UV exposure are a step in cancer development that does not rely on somatic mutations. These physiological events evidently include a disturbance of ribosome composition that percolates into widespread changes in protein translation and stress responses. The similarity between the actors involved in early UV-induced and spontaneous melanomas is therefore not a coincidence, if, as the results suggest, neonatal UV exposure acts on seemingly healthy variants to cause physiological changes that also lead to problems in ribosome regulation.

In trying to learn how a single neonatal UV dose causes melanoma, Ferguson et al. went from the environment, to the genome, and back again. These results shine a light on physiological changes that cause, rather than result from, the emergence of cancer. Still, why do transient, cell-wide changes in every cell of the population precede rare, permanent mutations arising in a founder cell? A clue is that the cancer phenotype can arise from non-mutational changes. For instance, melanocytes can become cancerous within four weeks of UV exposure if certain growth factors are expressed in the surrounding skin (Berking et al., 2004). Perhaps we have the paradigm backward when picturing driver mutations as inexorably pushing cells into becoming cancerous. Instead, the environment may be creating reversible physiological changes that temporarily ratchet the cell toward cancer; ‘driver' mutations may then merely act as pawls, locking in these changes within an occasional cell that then cannot revert to its healthy state when the environmental stress has ended.
